# Downregulations of AKT/mTOR Signaling Pathway for *Salmonella*-Mediated Suppression of Matrix Metalloproteinases-9 Expression in Mouse Tumor Models

**DOI:** 10.3390/ijms19061630

**Published:** 2018-05-31

**Authors:** Yu-Tzu Tsao, Chun-Yu Kuo, Shun-Ping Cheng, Che-Hsin Lee

**Affiliations:** 1 Division of Nephrology, Department of Medicine, Taoyuan General Hospital, Taoyuan 330, Taiwan; tsaoyutzu@gmail.com; 2 Institute of Clinical Medicine, National Yang-Ming University, Taipei 11221, Taiwan; 3 Graduate Institute of Basic Medical Science, School of Medicine, China Medical University, Taichung 404, Taiwan; jay999kimo@yahoo.com.tw; 4 Department of Physical Medicine and Rehabilitation, Taoyuan General Hospital, Ministry of Health and Welfare, Taoyuan 330, Taiwan; 5 Department of Biological Sciences, National Sun Yat-sen University, Kaohsiung 804, Taiwan; 6 Department of Medical Research, China Medical University Hospital, China Medical University, Taichung 404, Taiwan

**Keywords:** matrix metalloproteinases, metastasis, tumor-targeting, *Salmonella*

## Abstract

The roles of Matrix MetalloProteinases (MMPs), such as MMP-9, in tumor metastasis are well studied, and this in turns stimulates the development of MMP inhibitors as antitumor agents. Previously, *Salmonella* accumulation was observed in the metastatic nodules of the lungs after systemic administration. *Salmonella* significantly enhanced the survival of the pulmonary metastatic tumor-bearing mice. Based on our previous observation, we hypothesized that *Salmonella* could affect metastasis-related protein expression. The treatment of *Salmonella* clearly reduced the expression of MMP-9. Meanwhile, the MMP-9 related signaling pathways, including Phosph-Protein Kinase B (P-AKT) and Phosph-mammalian Targets Of Rapamycin (P-mTOR) were decreased after a *Salmonella* treatment. The *Salmonella* inhibited tumor cell migration by wound-healing and Transwell assay. The anti-metastatic effects of *Salmonella* were evaluated in mice bearing experimental metastasis tumor models. Consequently, *Salmonella* inhibited the expression of MMP-9 by reducing the AKT/mTOR pathway and metastatic nodules in vivo.

## 1. Introduction

Solid tumors frequently develop the microenvironment due to physiological stresses [1]. The expression of Matrix MetalloProteinase 9 (MMP-9) in tumors, which may be induced by a tumor microenvironment, contributes to a poor prognosis [2]. Much evidence has demonstrated that MMP-9 plays an important role in tumor growth and metastasis [3]. The MMP-9 as gelatinase is involved in the degradation of the extracellular matrix, angiogenesis, and the progression of tumors. The specific MMP-9 inhibitor is an active area of translational research for tumor treatment [4].

*Salmonella* is a Gram-negative facultative anaerobic rod that affects the gastrointestinal tract. *Salmonella* has been demonstrated as a tumor-targeting agent and has antitumor potential [5]. *Salmonella* exerts a variety of biological effects in antitumor responses, such as tumor-targeting, stimulating host immunity, chemosensitivity, and anti-angiogenesis [6,7,8]. However, the mechanism of *Salmonella* in the regulation of tumor metastasis is still unclear. Depending on our previous observation, *Salmonella* can reduce metastatic nodules and prolong the survival of mice in metastatic tumor models [9]. The aim of the present study was to characterize the mechanism of action of *Salmonella* on metastasis. The present study revealed that *Salmonella* inhibited cell migration of tumor cells via the Phosph-Protein Kinase B (P-AKT) and Phosph-mammalian Targets Of Rapamycin (P-mTOR) signaling pathways.

## 2. Results

### 2.1. Salmonella Inhibition of Tumor Cell Migration

**Figure 1:**
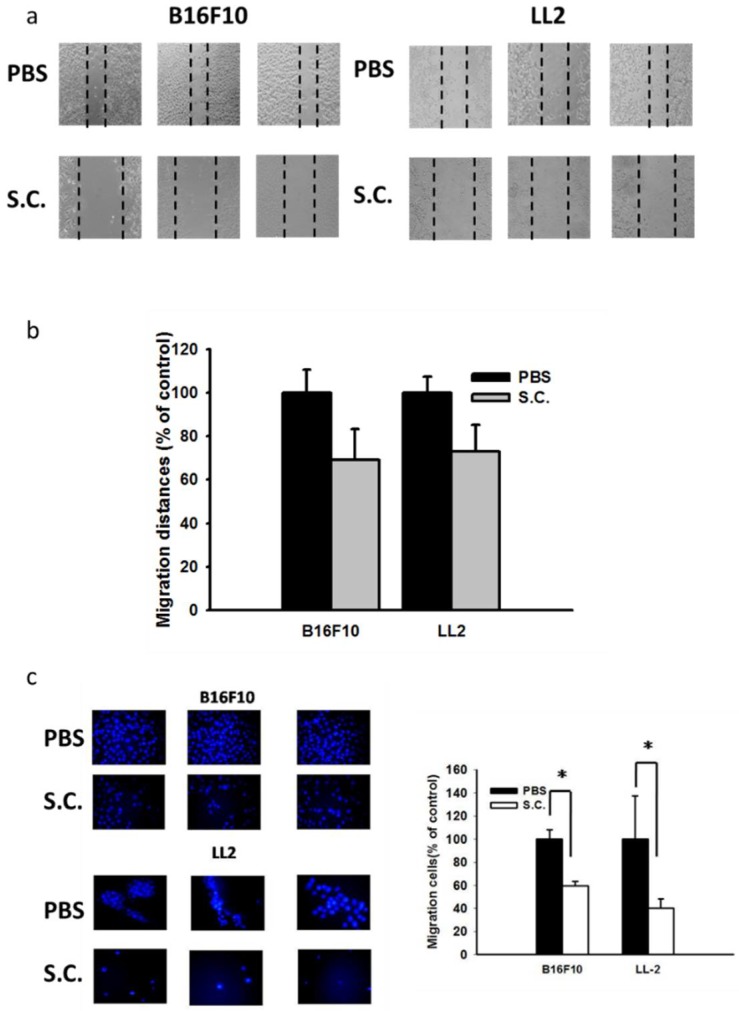
Effects of *Salmonella* (S.C.) on cellular migration in B16F10 and LL2 cells. B16F10 cells and LL2 cells (2 × 10^5^ cells/well) were placed onto plates and incubated at 37 °C for 24 h. The cells cocultured with *Salmonella* (Multiplicity Of Infection (MOI) = 10) for 4 h. The migration distances of different groups of B16F10 and LL2 cells were shown (**a**) and measured (**b**) (×400); (**c**) Tumor cells were placed on the upper layer of a cell culture insert with permeable membrane and then treated with *Salmonella* (MOI = 10) for 4 h. Following an incubation 24 h, the cells migrated through the membrane were stained with 4′,6-Diamidino-2-Phenylindole (DAPI) and counted under a fluorescence microscope (×400).

The B16F10 mouse melanoma and LL2 mouse lung tumor cells, both treated with *Salmonella*, were tested for their ability to inhibit the migration of tumor cells. By using the wound–healing assay, the migration of B16F10 cells was significantly decreased upon the addition of *Salmonella* compared with that of phosphate buffered saline (PBS) groups. Meanwhile, *Salmonella* reduced the migration of LL2 cells (Figure 1a,b). Although the migration of tumor cells treated with *Salmonella* was observed by using the wound-healing assay, it did not preclude the possibility of cellular proliferation. Indeed, the migration of the tumor cells, including B16F10 and LL2 cells was significantly reduced after a *Salmonella* treatment by using the Transwell assay (Figure 1c). The results suggested that *Salmonella* reduced the migration of tumor cells.

### 2.2. The Phospho-Protein Kinase B (P-AKT)/Phospho-Mammalian Targets of the Rapamycin (P-mTOR) Pathway as Requirement for Salmonella-Mediated MMP-9 Expression

**Figure 2:**
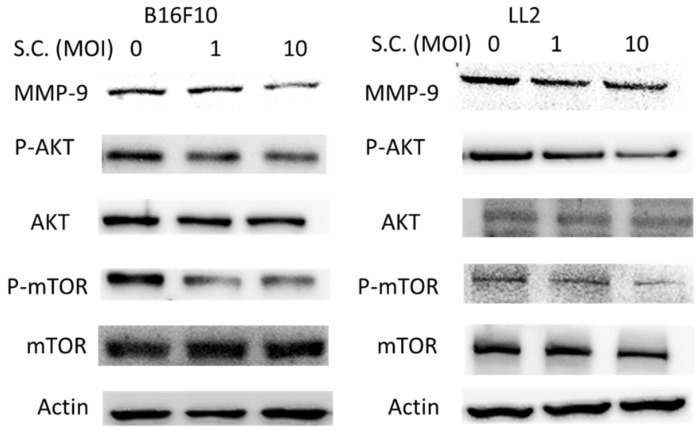
Effects of *Salmonella* (S.C.) on Matrix MetalloProteinase 9 (MMP-9) expression in B16F10 and LL2 cells. B16F10 cells and LL2 cells (2 × 10^5^ cells/well) were placed into plates and incubated at 37 °C for 24 h. The cells cocultured with *Salmonella* (MOI = 1–10) for 4 h. The protein expression of B16F10 and LL2 cells was measured.

**Figure 3:**
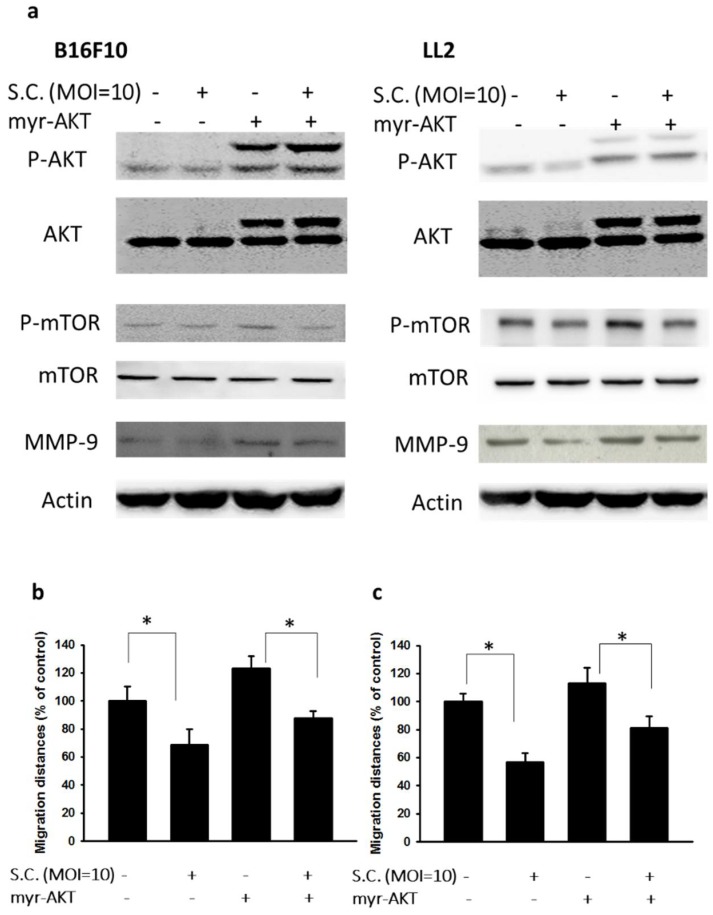
Constitutively active- Phosph-Protein Kinase B (P-AKT) reduced *Salmonella* (S.C.)-induced decrease of MMP-9. The B16F10 and LL2 cells were transfected with constitutively active AKT plasmids for 16 h prior to 4 h *Salmonella* treatment. (**a**) The expression of phosphorylation AKT, AKT, phosphorylation Phosph-mammalian Targets Of Rapamycin (P-mTOR), mTOR, and MMP-9 protein in B16F10 and 4T1 cells was determined. The immunoblotting assay was repeated three times with similar results. The migration distances of B16F10 (**b**) and LL2 (**c**) cells were measured. (*n* = 3, data are mean ± SD. * *p* < 0.05).

*Salmonella* can regulate the migration of tumor cells. As expected, *Salmonella* may affect the enzyme responsible for tumor migration, such as MMP-9. B16F10 cells and LL2 cells were treated with *Salmonella* (Multiplicity Of Infection (MOI) = 0–10) and the expression of MMP-9 in tumor cells was measured. The AKT/mTOR axis was involved in the expression of MMP-9 in tumors. The Phosphatidylinositol 3-Kinase (PI3K) inhibitors suppressed MMP-9 expression, suggesting that the PI3K/AKT pathways are involved in MMP-9 regulation [10]. The protein expressions of phospho-AKT/mTOR were examined in two cell lines treated with *Salmonella* (Figure 2). We found that *Salmonella* did in fact reduce the expression of MMP-9 in a dose-dependent manner (Figure 2). Meanwhile, the expression of phospho-AKT/phospho-mTOR was reduced after the *Salmonella* infection. Regarding the involvement of the AKT/mTOR axis in the *Salmonella*-mediated suppression of MMP-9, we were surprised to learn that cells were transfected with constitutively active AKT plasmids. Determination of the Western blotting showed that transfection with constitutively active AKT plasmid in tumor cells resulted in an inversion of *Salmonella*-regulated MMP-9 expression in tumor cells (Figure 3a). The migration distance of tumor cells was suppressed after *Salmonella* treatment, as previous described. The migration distances of B16F10 and LL2 cells treated with *Salmonella* could be reversed after constitutively active AKT plasmid transfection (Figure 3b,c). The results indicated that AKT/mTOR signaling pathway might play the role in the *Salmonella*-regulated decrease of MMP-9 and tumor cell migrated behavior.

### 2.3. Salmonella Supresssion of Matrix MetalloProteinase 9 Expression In Vivo

**Figure 4:**
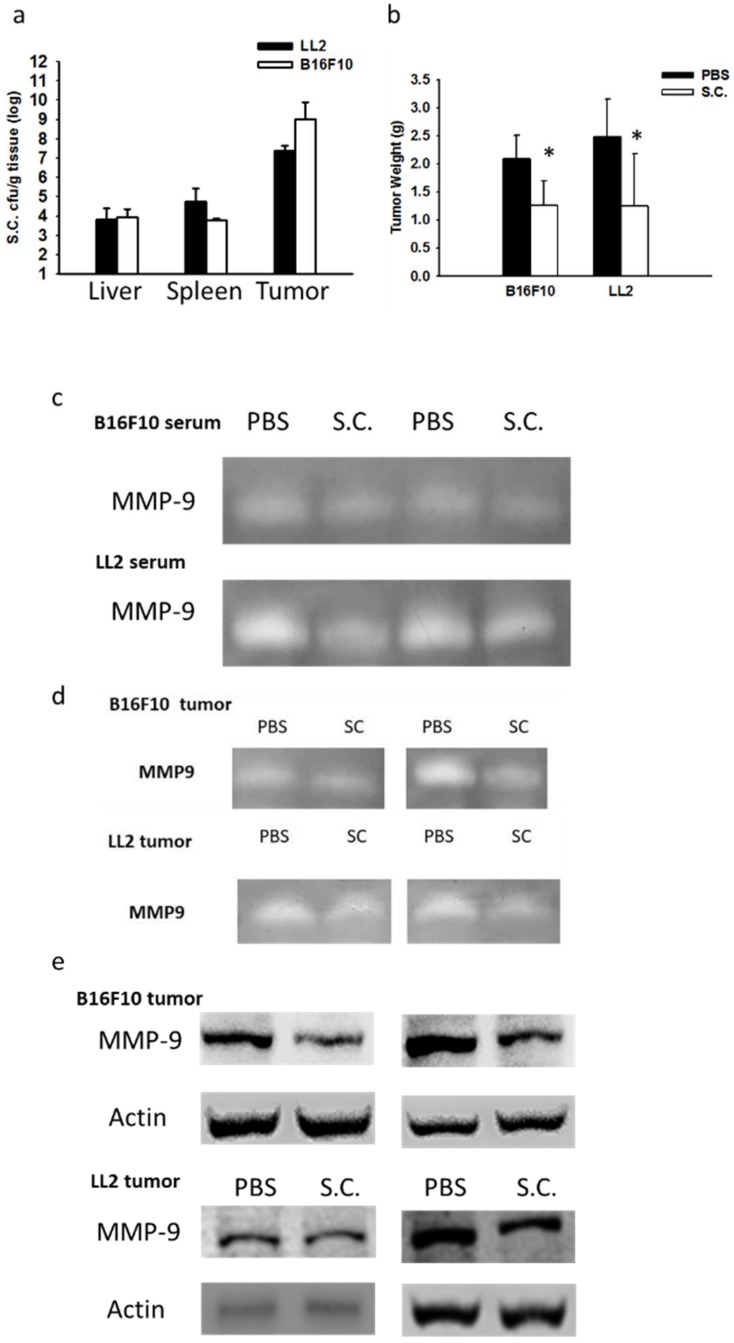
*Salmonella* (S.C.) reduced the function and expression of MMP-9 in vivo. The C57BL/6 mice had been subcutaneously inoculated with B16F10 and LL2 cells at day 0, and the tumor bearing mice were intraperitoneal injected with *Salmonella* (10^6^ cfu) at day 7. At day 10, the mice were sacrificed. The serum, tumors, livers, and spleen were collected. (**a**) The amounts of accumulated *Salmonella* in organs was determined; (**b**) The antitumor activity of *Salmonella* was determined by tumor weight. (*n* = 4, data are mean ± SD. * *p* < 0.05); The enzyme activity of MMP-9 in serum (**c**) and tumors (**d**) was determined by gelatin zymography; (**e**) The protein expression of MMP-9 in tumors derived from *Salmonella*-treated mice or control mice was determined by Western blotting.

We obtained similar results demonstrating that the tumor-targeting potential of *Salmonella* and anti-tumor activity of *Salmonella* in two tumor models (Figure 4a,b). Tumors can digest extracellular matrixes to facilitate their migration to secondary sites by releasing MMP-9 themselves [11]. Using gelatin zymography to detect MMP-9 activity is required. To verify the study in vivo, tumor-bearing mice were treated with *Salmonella* (10^6^ colony-forming units (cfu)) and sacrificed after 3 days. Serum and tumor tissues were collected and analyzed by gelatin zymography and Western blotting (Figure 4c–e). *Salmonella* reduced the function of MMP-9 in serum and tumors from tumor-bearing mice (Figure 4c,d). By using Western blotting analysis, the reduced protein expressions of MMP-9 in two tumor models were observed in *Salmonella*-treated group (Figure 4e) These findings suggested that *Salmonella* inhibited the expression and activity of MMP-9 in vivo.

### 2.4. Salmonella Inhibition of Tumor Metastasis In Vivo

**Figure 5:**
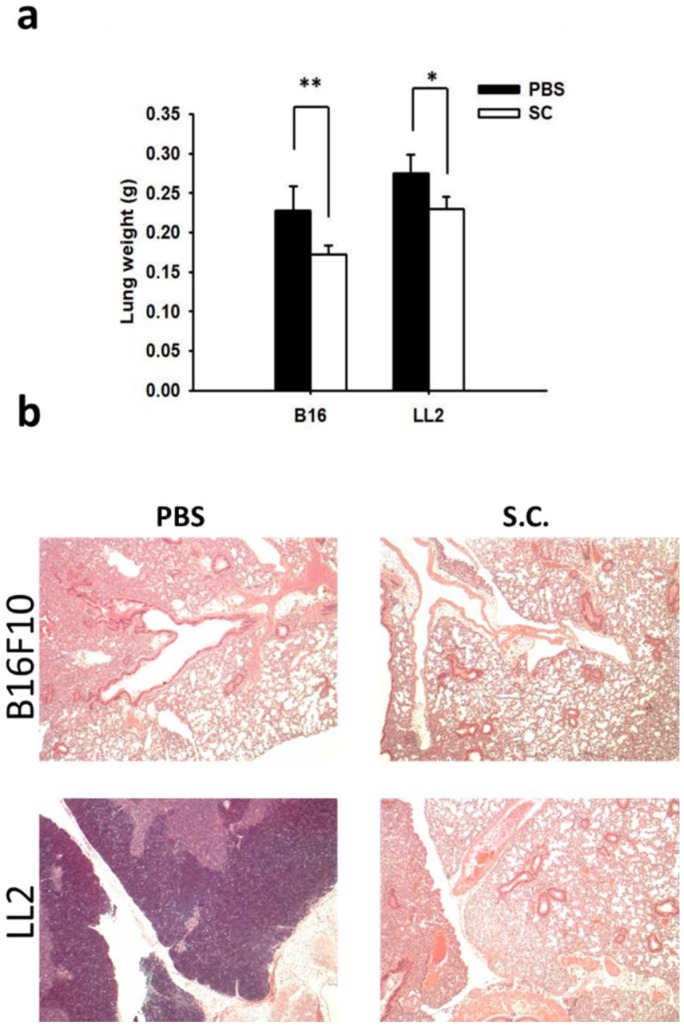
*Salmonella* (S.C.) inhibited metastasis in vivo. The C57BL/6 mice were injected with B16F10 and LL2 cells (10^5^) pre-incubated with or without *Salmonella* (MOI = 10) for 4 h via the tail vein at day 0. (**a**) At day 20, the wet lung weight of the lungs was measured. (*n* = 3, data are mean ± SD. * *p* < 0.05; ** *p* < 0.01). (**b**) The metastatic pulmonary nodules were observed after the intravenous injection of cells (×400).

On our previous study, we established that mice injected with tumor cells admixed with metastatic inducers or metastatic inhibitors to identify the activity of anti-metastatic molecules [12]. We now want to know whether *Salmonella* could inhibit metastasis: the tumor cells either pre-incubated with *Salmonella* or not were injected into mice via tail vein. *Salmonella* affected the tumor mass in lungs (Figure 5a). There are more B16F10 tumor nodules in the mice injected with cells than those in the mice injected cells admixed with *Salmonella* (Figure 5b). The similar results were obtained in LL2 tumor models. Taken together, these results demonstrated that *Salmonella* affects tumor metastasis in vivo.

## 3. Discussion

Tumor-target therapy of *Salmonella* is attracting increasingly more attention for researchers and is considered to be a new strategy against solid tumors [8,13,14,15]. When *Salmonella* is used as an antitumor agent, it has many features for inhibiting tumor growth. Previously, we found that *Salmonella* not only targeted primary tumors, but also small metastatic tumors [16]. The results imply that *Salmonella* could reduce tumor metastasis. Observing the expression of MMP-9 protein in both tumor cells treated with *Salmonella* were significantly decreased, which was consistent with the result of the cell migration ability detected by wound-healing and Transwell assay. In animal studies, the less metastatic nodules and lung weights were observed in *Salmonella*-administrated groups.

Increasingly more evidence has suggested that cancer stem cells contribute to metastasis [17]. The MMP-9 is one of the cancer stem cell markers [18]. In this study, *Salmonella* reduced the expression of MMP-9 via the AKT/mTOR pathway. *Salmonella* had been demonstrated to inhibit tumor growth by targeting the cancer stem cell niche [19]. The hypoxic regions of the tumor are a privileged site in which the *Salmonella* as well as the cancer stem cells reside; therefore, the *Salmonella* is able to aim at the at the cancer stem cell [19]. The results are consistent with previous reports. The accumulation of *Salmonella* in tumor sites has been confirmed in various solid tumors, including breast cancer, melanoma, bladder cancer, liver cancer, lung cancer, and colon cancer [9,16,19,20]. Although *Salmonella* has enormous potential for targeting solid tumors, the mechanisms are largely unknown. *Salmonella* may use enhanced permeability and retention effect to specifically target to tumor sites [21]. In addition, host immune cells in healthy organs cleared *Salmonella* more rapidly than those in a tumor microenvironment, where immune sites are privileged [22,23]. Moreover, the nutrients and hypoxia regions in tumors may attract *Salmonella* to target the tumor sites [9].

This study helps to better understand how the interaction between *Salmonella* and tumor microenvironment. *Salmonella* may reduce the modification of a second organ microenvironment by inhibiting the secretion and function of MMP-9 from a primary tumor. *Salmonella* influences oncoproteins—such as hypoxia-inducible factors, indoleamine 2,3-dioxygenase, and *p*-glycoprotein—through the AKT/mTOR signaling pathways in tumor cells [6,24,25]. The cellular AKT/mTOR axis plays an important role in cellular physiology and homeostasis [26]. Many small molecular drugs target this axis and contribute to control the tumor growth [27]. Autophagy is initiated in response to cellular stress, including *Salmonella* infection. *Salmonella* triggered cell autophagy through *Salmonella*-induced amino acid starvation [25,26]. Autophagy can regulate protein synthesis [28]. The AKT/mTOR pathway is involved in a negative regulator of autophagy [29]. A *Salmonella* infection could influence the AKT/mTOR signaling pathways in cells [30]. *Salmonella* induces autophagy by decreasing AKT/mTOR signaling pathway [29,30]. In our system, the MMP-9 is downregulated through *Salmonella*, which decreases AKT/mTOR signaling pathway. *Salmonella* has a tumor-targeting potential and inhibits the AKT activity, implying that *Salmonella* suppresses tumor growth through inhibiting the AKT/mTOR signal pathway.

## 4. Materials and Methods

### 4.1. Cells, Reagents, Animal, Bacteria, and Plasmids

Murine melanoma cells (B16F10) and murine lung carcinoma (LL2) cells were cultured in Dulbecco’s Modified Eagle’s Medium (DMEM), containing 10% of fetal bovine serum and 50 μg/mL gentamicin at 37 °C in 5% CO_2_. The 4′,6-Diamidino-2-Phenylindole (DAPI) and gelatin were purchased from Sigma-Aldrich (Sigma Aldrich, St. Louis, MO, USA). C57BL/6 mice were purchased from the National Laboratory Animal Center of Taiwan. The experimental protocol was approved by the Laboratory Animal Care and Use Committee of the National Sun Yat-sen-University (permit number: 10635, 20 December 2017). The *Salmonella* and the constitutively active AKT plasmid were previously described [9,24].

### 4.2. Wound-Healing and Transwell Assay

The culture inserts (IBIDI, Martinsried, Germany) plated on 24 well plates were used to measure the wound-healing according to the manufacturer’s instruction. The migration distance was measured after 24 h using a microscope. The migration distances of untreated tumor cells were set to 100% and were compared with cells treated with *Salmonella* for 4 h. The Transwell cultures (ThermoFisher Scientific, Waltham, MA, USA) plated on 24 well plates were used to observe the cell migration according to the manufacturer’s instruction. The migration cells were stained with DAPI and counted under fluorescence microscope. The number of migration cells of untreated tumor cells were set to 100% and were compared with cells treated with *Salmonella* for 4 h.

### 4.3. Western Blotting, Gelatin Zymography, and Transfection

The Bicinchoninic Acid (BCA) protein assay (Pierce Biotechnology, Rockford, IL, USA) was used to determine the protein contents. The SDS-PAGE was used to fractionate the protein samples. Then, protein samples were transferred onto hybond-enhanced chemiluminescence nitrocellulose membranes (Pall Life Science, Glen Cove, NY, USA). The membranes were probed with various antibodies, such as MMP-9 (Santa Cruz Biotechnology, Santa Cruz, CA, USA), phosphorylation-AKT (Santa Cruz Biotechnology), AKT (Santa Cruz Biotechnology), phosphorylation-mTOR (Cell Signaling, Danvers, MA, USA), mTOR (Cell Signaling), or β-actin (Sigma-Aldrich). The appropriate horseradish peroxidase-conjugated antibodies were used as secondary antibodies. The protein-antibody complexes were visualized by enhanced chemiluminescence system (T-Pro Biotechnology, New Taipei City, Taiwan). A 7.5% acrylamide gel containing gelatin was used to separate protein. Then, the gel was stained with a staining solution for 1 h and was washed with destaining solution until bands could clearly be seen. Tumor cells were transfected with the constitutively active AKT plasmid, using Lipofectamine 2000. At post-transfection, cells were treated with *Salmonella* for 4 h or not. The cell lysates were then harvested.

### 4.4. Mouse Experiments

The C57BL/6 mice was subcutaneously inoculated with B16F10 (10^6^) and LL2 (10^6^) cells at day 0, and the tumor bearing mice were intraperitoneal injected with *Salmonella* (10^6^ cfu) at day 7. At day 10, the mice were sacrificed. The serum, tumors, livers, and spleen were collected and weighed, and the number of *Salmonella* was counted on Lysogeny broth plates. MMP-9 in serum and tumors was determined by gelatin zymography and Western blotting. In a parallel experiment, mice were injected with B16F10 (10^6^) and LL2 cells (10^5^) pre-incubated with or without *Salmonella* (MOI = 10) for 4 h via the tail vein at day 0. At day 20, tumor-bearing mice were sacrificed, and the lungs were removed, weighed, and histologically examined.

### 4.5. Statistical Analysis

The significant differences between groups were determined by Analysis of variance (ANOVA). Any *p* value less than 0.05 is considered statistically significant.

## 5. Conclusions

*Salmonella* has an advantage over other antitumor agents because *Salmonella* could target to tumor sites [31]. *Salmonella* preferentially accumulated not only in primary tumors but also in metastatic nodules. In this study, *Salmonella* reduced tumor migration in vitro and the formation of tumor nodules in vivo by inhibiting MMP-9 expression. Thus, *Salmonella*-mediated tumor therapy should attract more attention in the future.

## Abbreviations

MMP	Matrix metalloproteinases
AKT	Protein kinase B
mTOR	Mammalian targets of rapamycin
PI3K	Phosphatidylinositol 3-kinase
S.C.	Salmonella
MOI	The multiplicity of infection
DAPI	4′,6-Diamidino-2-phenylindole
cfu	Colony-forming units
